# Genome-wide identification and characterization of ATP-binding cassette transporters in the silkworm, *Bombyx mori*

**DOI:** 10.1186/1471-2164-12-491

**Published:** 2011-10-07

**Authors:** Shumin Liu, Shun Zhou, Ling Tian, Enen Guo, Yunxia Luan, Jianzhen Zhang, Sheng Li

**Affiliations:** 1 Key Laboratory of Developmental and Evolutionary Biology, Institute of Plant Physiology and Ecology, Shanghai Institutes for Biological Sciences, Chinese Academy of Sciences, Shanghai 200032, China

## Abstract

**Background:**

The ATP-binding cassette (ABC) transporter superfamily is the largest transporter gene family responsible for transporting specific molecules across lipid membranes in all living organisms. In insects, ABC transporters not only have important functions in molecule transport, but also play roles in insecticide resistance, metabolism and development.

**Results:**

From the genome of the silkworm, *Bombyx mori*, we have identified 51 putative ABC genes which are classified into eight subfamilies (A-H) by phylogenetic analysis. Gene duplication is very evident in the ABCC and ABCG subfamilies, whereas gene numbers and structures are well conserved in the ABCD, ABCE, ABCF, and ABCH subfamilies. Microarray analysis revealed that expression of 32 silkworm ABC genes can be detected in at least one tissue during different developmental stages, and the expression patterns of some of them were confirmed by quantitative real-time PCR. A large number of ABC genes were highly expressed in the testis compared to other tissues. One of the ABCG genes, *BmABC002712*, was exclusively and abundantly expressed in the Malpighian tubule implying that *BmABC002712 *plays a tissue-specific role. At least 5 ABCG genes, including *BmABC005226*, *BmABC005203*, *BmABC005202*, *BmABC010555*, and *BmABC010557*, were preferentially expressed in the midgut, showing similar developmental expression profiles to those of 20-hydroxyecdysone (20E)-response genes. 20E treatment induced the expression of these ABCG genes in the midgut and RNA interference-mediated knockdown of *USP*, a component of the 20E receptor, decreased their expression, indicating that these midgut-specific ABCG genes are 20E-responsive.

**Conclusion:**

In this study, a genome-wide analysis of the silkworm ABC transporters has been conducted. A comparison of ABC transporters from 5 insect species provides an overview of this vital gene superfamily in insects. Moreover, tissue- and stage-specific expression data of the silkworm ABCG genes lay a foundation for future analysis of their physiological function and hormonal regulation.

## Background

The ATP-binding cassette (ABC) transporters form one of the largest family of membrane proteins [1]. With 48 members in total, the ABC transporter family from animals was first identified in the human genome [2]. Ranging from 28 to ~200 members, the ABC transporter family is present in all organisms [3]. Based on sequence similarity of the ATP-binding sites, the 48 human ABC transporters can be classified into seven subfamilies (A to G, ABCA to ABCG). The eighth subfamily (H) was defined following the analysis of the genome of the fruitfly, *Drosophila melanogaster *(Diptera) [2]. In addition to the fruitfly [2], the ABC transporters have been previously analyzed in another insect species, the mosquito, *Anopheles gambiae *(Diptera), at the genome-wide level [4].

The ABC transporters share highly conserved domains known as nucleotide binding domains (NBDs). Each NBD contains three characteristic motifs, including Walker A box, Walker B box, and ABC signature C which links the two Walker boxes [5]. NBD binds and hydrolyses ATP and provides energy to transport molecules against concentration gradients. In addition to NBD, a eukaryotic ABC transporter usually consists of one or two transmembrane domains (TMD). The ABC transporters which have two NBDs and two TMDs are called full transporters, whereas those with one NBD and one TMD are called half transporters, which often constitute a functional unit by forming a homo- or heterodimer [6].

According to their functions, the ABC proteins can be classified as importers, exporters and non-transport proteins [7]. Importers and exporters are responsible for transport of a wide variety of substances, whereas the third class of ABC proteins are apparently not involved in molecule transport but in cellular processes such as DNA repair, translation or regulation of gene expression [8]. In human, known functions of ABC transporters include cholesterol and lipid transport, multidrug resistance, antigen presentation, mitochondrial iron homeostasis, and the ATP-dependent regulation of ion channels. Mutations in ABC genes have been associated with a range of disorders, including cystic fibrosis, hypercholesterolemia and diabetes [9]. In insects, it has been shown that ABC transporters have roles in uric acid metabolism, development and possibly in insecticide resistance [10]. Due to the importance of ABC transporters, several members of ABC transporters have been extensively studied in several model insects, including the fruitfly and the silkworm, *Bombyx mori *(Lepidoptera). One of the best studied insect ABC transporters is White, which is a typical ABCG transporter involved in pigment transport in insect eyes [11,12].

In a microarray study published previously [13,14], we detected multiple silkworm ABC genes exhibiting possible regulation by the molting hormone, 20-hydroxyecdysone (20E). To this end, we decided to identify and characterize the silkworm ABC transporters at the genome-wide level. Very recently, three silkworm ABC transporter subfamilies, including ABCB, ABCC and ABCG, were analyzed regarding to their possible xenobiotic resistance [10]. In this study, we have identified 51 putative silkworm ABC genes in total and analyzed their phylogenetic relationships. Moreover, we have investigated their temporal and spatial expression patterns using microarray and quantitative real-time PCR (qRT-PCR), with focus on the ABCG subfamily.

## Results and Discussion

### Identification of the silkworm ABC transporters

The NBDs of all the *D. melanogaster *ABC transporters were used as queries for BLASTP search against the two silkworm genome databases, SilkDB and KAIKObase. Each potential silkworm ABC transporter was validated by searching its known orthologs from the protein database of NCBI and further searching its NBD and TMD with the Pfam program. As a result, a total of 51 putative ABC genes were identified in the silkworm genome. A previous study has identified 56 ABC genes in *D. melanogaster *[2]. For comparative analysis, we also identified 68 ABC genes from the flour beetle, *Tribolium castaneum *(Coleoptera), 52 from *A. gambiae *(8 more compared to 44 in [4] due to the updated mosquito genome), and 43 from the honeybee, *Apis mellifera *(Hymenoptera) (Table 1).

**Table 1:** Subfamilies of ABC genes in 5 insect species, *Bombyx mori*, *Drosophila melanogaster, Tribolium castaneum, Anopheles gambiae*, and *Apis mellifera *as well as the human, *Homo sapiens*.

ABC subfamily	*Bombyx mori*	*Drosophila melanogaster*	*Anopheles gambiae*	*Tribilium castaneum*	*Apis mellifera*	*Homo sapiens*
A	6	10	9	9	3	13

B	8	8	5	6	7	11

C	15	14	13	31	9	12

D	2	2	2	2	2	4

E	1	1	1	1	1	1

F	3	3	3	3	3	3

G	13	15	16	13	15	5

H	3	3	3	3	3	0

**Total**	**51**	**56**	**52**	**68**	**43**	**48**

In SilkDB, 37 ABC genes have evidences of mRNA expression with EST sequences, which were collected from 36 cDNA libraries of multiple tissues during different developmental stages. Based on the EST information, *BmABC010129 *shows the highest transcript level with 26 hits. The largest ABC transporter (1794 amino acids) and the smallest one (280 amino acids) are encoded by *BmABC012789 *and *BmABC010825*, respectively (Table 2).

**Table 2:** Summary of the 51 ABC genes identified in the silkworm genome.

subfamily	*Gene*	Size(AA)	**Chr**.	Scaffold	Position	Exon	EST	probe
A	*BmABC009503*	1040	14	nscaf2953	1661521..1682998	21	2	sw18797

A	*BmABC012789*	1794	16	nscaf3058	7588846..7625968	34	1	sw17785

A	*BmABC007217*	1028	17	nscaf2873	124005..140286	16	1	sw08704

A	*BmABC007221*	1747	17	nscaf2873	43128..67099	24	6	sw19260

A	*BmABC007218*	474	17	nscaf2873	71197..79589	9	1	sw19450

A	*BmABC004187*	452	19	nscaf2770	824533..837264	8	0	sw05280

B	*BmABC000725*	1149	1	nscaf1690	6582863..6601286	22	2	sw20470

B	*BmABC000724*	1307	1	nscaf1690	6553161..6573490	26	3	sw08035

B	*BmABC005473*	850	8	nscaf2828	3932738..3950945	16	4	sw12680

B	*BmABC009452*	1312	14	nscaf2953	1939875..1966564	22	0	sw01511

B	*BmABC007494*	1268	15	nscaf2887	1945690..1965221	23	3	sw11440

B	*BmABC004142*	581	19	nscaf2767	4285226..4299688	13	2	sw11283

B	*BmABC012743*	702	22	nscaf3056	487542..500932	12	12	sw08227

B	*BmABC011228*	1311	23	nscaf3026	3885423..3912497	27	2	sw13612

C	*BmABC006882*	1334	10	nscaf2859	1597393..1617896	21	1	sw11399

C	*BmABC010332*	1180	12	nscaf2990	12995..30206	18	1	sw20821

C	*BmABC010331*	496	12	nscaf2990	39940..44300	6	1	sw18980

C	*BmABC010636*	1594	12	nscaf2998	305046..322760	22	0	sw05268

C	*BmABC010330*	1251	12	nscaf2990	44879..78780	19	1	sw12816

C	*BmABC007793*	305	15	nscaf2888	999476..1002597	6	0	sw12273

C	*BmABC007738*	983	15	nscaf2888	908517..930623	18	6	sw20863

C	*BmABC007769*	297	15	nscaf2888	134904..141029	7	2	sw20393

C	*BmABC007785*	728	15	nscaf2888	767551..792765	14	4	sw02050

C	*BmABC007735*	1364	15	nscaf2888	1007371..1038895	22	11	sw07429

C	*BmABC007784*	540	15	nscaf2888	751792..764508	9	1	sw04125

C	*BmABC003359*	760	15	nscaf2655	606966..624399	14	0	sw09343

C	*BmABC007792*	658	15	nscaf2888	984948..994967	12	0	sw19077

C	*BmABC011220*	1263	23	nscaf3026	4262367..4292000	29	2	sw18613

C	*BmABC010849*	779	UN	nscaf3004	47746..61144	13	2	sw20170

D	*BmABC012688*	503	22	nscaf3055	13802..26162	12	1	sw12200

D	*BmABC004616*	754	27	nscaf2801	381693..398156	14	0	sw19711

E	*BmABC010129*	608	7	nscaf2986	3437097..3447398	12	26	sw13221

F	*BmABC002004*	622	1	nscaf2210	4610405..4619608	11	17	sw08321

F	*BmABC006964*	638	10	nscaf2860	2849092..2861275	10	1	sw09644

F	*BmABC007869*	906	15	nscaf2888	4038262..4052670	18	9	sw19088

G	*BmABC002581*	498	5	nscaf2529	4518502..4529267	7	0	sw13270

G	*BmABC002712*	312	5	nscaf2529	4492836..4497700	6	0	sw15243

G	*BmABC002922*	690	10	nscaf2575	3237764..3274876	12	6	sw05247

G	*BmABC002924*	458	10	nscaf2575	3311835..3326257	8	0	sw07486

G	*BmABC012035*	585	11	nscaf3034	3590330..3612716	8	2	sw03333

G	*BmABC005226*	647	12	nscaf2826	44749..62984	11	4	sw13968

G	*BmABC005203*	530	12	nscaf2825	7374..22039	8	3	sw15660

G	*BmABC005202*	701	12	nscaf2825	75958..87920	11	1	sw05618

G	*BmABC010555*	598	12	nscaf2993	5875864..5891335	11	4	sw17556

G	*BmABC010557*	470	12	nscaf2993	5970529..5979789	8	0	NO

G	*BmABC000220*	823	22	nscaf1681	5597989..5616076	14	0	sw08011

G	*BmABC000472*	640	22	nscaf1681	5666445..5676633	13	0	sw08685

G	*BmABC005094*	727	25	nscaf2823	124639..134335	8	0	sw12687

H	*BmABC010726*	791	26	nscaf3003	3211528..3248514	15	6	sw05988

H	*BmABC010825*	280	26	nscaf3003	3337089..3347904	7	1	sw18772

H	*BmABC010760*	607	26	nscaf3003	7482864..778388	10	1	sw04232

### Phylogenetic analysis of the silkworm ABC transporters

**Figure 1:**
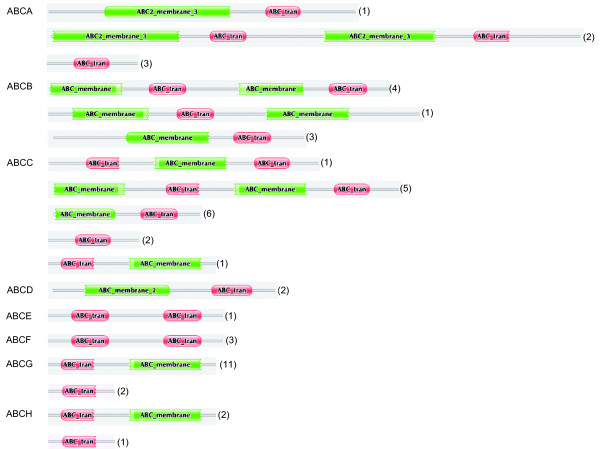
**Conserved domains of the silkworm ABC transporters**. Red and green show nucleotide binding domain (NBD; ABC_tran) and transmembrane domain (TMD; ABC_membrane), respectively. The program pfam was used to identify NBD and TMD domains of each silkworm ABC transporter.

**Figure 2:**
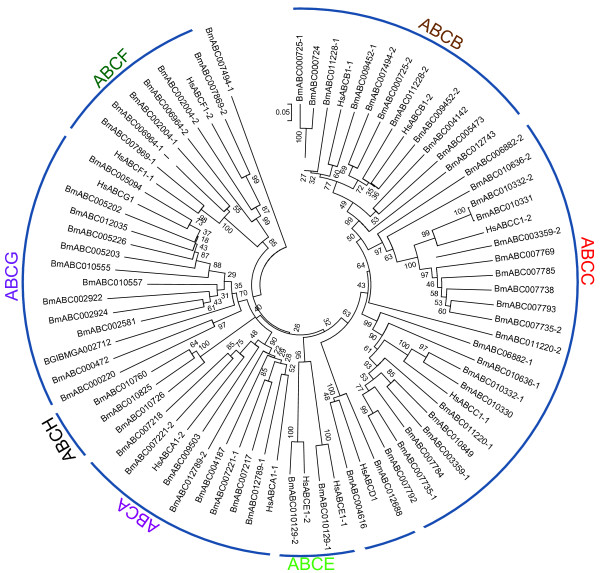
**Phylogenetic tree of the silkworm ABC transporters**. The phylogenetic tree was constructed using a neighbor-joining technique to analyze the amino acid sequences of the nucleotide binding domain (NBD). Analysis was performed with the program package MEGA4.0. The number at the branch point of the node represents the value resulting from 1000 replications and gaps were deleted with pairwise deletion method.

Each silkworm ABC transporter possesses one or two conserved NBDs (Figure 1). We performed a multiple sequence-alignment to construct a phylogenetic tree on the basis of NDBs of the 51 silkworm ABC transporters. Phylogenetic analysis revealed that silkworm ABC transporters can be divided into 8 subfamilies (A-H) with high bootstrap values. The ABCC subfamily can be separated into 2 groups, one of which shares high similarity to the ABCB subfamily (Figure 2). Among the 51 silkworm ABC transporters, there are 11 full transporters and 26 half transporters. The ABCA, ABCB and ABCC subfamilies contain full transporters, whereas half transporters exist in 6 subfamilies rather than ABCE and ABCF (Figure 1). The 4 ABCE and ABCF proteins lack of TMDs. One putative ABCB transporter contains one NDB and two TMDs, while one putative ABCC transporter contains two NDBs and one TMD. In addition, we found that 8 silkworm genes encode obvious NDBs show high similarity to ABC genes in the silkworm and other organisms. For convenience, we referred the 10 putative ABC proteins as incomplete ABC proteins. The incomplete sequences of those ABC genes might be because the genome of silkworm is not at completion and annotation of the entire genome is not complete. For comparative analysis, we also constructed phylogenetic trees for the ABC transporters from *T. castaneum *(Additional File 1: Fig. S1), *A. gambiae *(Additional File 2: Fig. S2), and *A. mellifera *(Additional File 3: Fig. S3). Similarly, the ABC transporters in each insect species can be divided into 8 subfamilies (A-H) (Table 1).

### Genomic distribution of the silkworm ABC transporters

Of the 51 silkworm ABC genes, 50 are dispersed on 17 chromosomes and 1 on an unmapped scaffold. Gene structures of the 51 silkworm ABC transporters show high complexity with exon numbers ranging from 6 to 34 (Table 2). There are two major gene clusters located on chromosomes 12 and 15, which contain 9 and 10 ABC genes, respectively. In addition, chromosomes 10 and 22 each contain 4 ABC genes. Gene duplication is very evident in chromosomes 12 and 15, also occurs in chromosomes 10, 17 and 26 by tandem duplication (Additional File 4: Fig. S4).

### Characterization of the silkworm ABC transporter subfamilies

Since the insect ABC transporters can be classified into 8 subfamilies (A-H), we characterized each subfamily in the silkworm and four other insect species for comparison.

#### ABCA

The ABCA transporters in mammals perform critical functions in the control of cellular lipid-transport processes [15], yet little is known about their physiological functions in insects. Among the 6 silkworm ABCA genes, *BmABC007217*, *BmABC007218 *and *BmABC007221 *are closely located on chromosome 17. The silkworm ABCA subfamily consists of 2 full transporters, 1 half transporter, and 3 incomplete ABC proteins containing one single NBD in each member. This subfamily includes two of the largest ABC transporters encoded by *BmABC012789 *(1794 amino acids) and *BmABC007221 *(1747 amino acids). In human, all ABCA transporters are full transporters [2]. In the plant, *Arabidopsis thaliana*, 11 are half transporters and only one is a full transporter [16]. No ABCA transporters have been identified in the yeast, *Saccharomyces cerevisiae *[17]. Interestingly, all the 9 ABCA transporters in the mosquito, *A. gambiae*, are full transporters, whereas both full and half transporters exist in the ABCA subfamily of the four other insect species, indicating that the ABCA subfamily varies greatly during evolution in insects and other organisms. In addition, phylogenetic analysis reveals that 3 insect ABCA transporters, *BmABC012789*, *AGAP010416 *and *TcABCA2*, are closely related (bootstrap value of 99%), implying that they are orthologs (Additonal File 5: Fig.S5).

#### ABCB

ABCB1 was the first human ABC transporter to be cloned and characterized through its function to confer a multidrug resistance (MDR) phenotype to cancer, and later studies revealed several more MDRs in the ABCB subfamily [18]. Based on sequence similarity, the insect ABCB transporters are thought to be involved in resistance to insecticides and other chemicals [10]. Among the 8 silkworm ABCB genes, *BmABC000725 *and *BmABC000724 *are closely distributed on chromosome 1 suggesting they were arisen by tandem duplication. The hypothesis of gene duplication is supported by phylogenetic analysis of the ABCB subfamily, which indicates that the two genes are closely related in evolution (Additonal File 6: Fig. S6). The silkworm ABCB subfamily consists of 4 full transporters, 3 half transporters, and 1 incomplete ABC protein. The same as other organisms, the ABCB subfamily in all the 5 insect species consists of both full and half transporters. The *D. melanogaster ABCB *gene, *Mdr49*, has multiple functions. Disruption of *Mdr49 *results in change of sensitivity to colchicines [19]. *Mdr49 *is not only involved in the transport of polycyclic aromatic hydrocarbons [20], but also controls germ cell attractant [21]. The ABCB transporter encoded by *BmABC009452 *is ~49% identical to that of Mdr49 in sequence (Additonal File 6: Fig. S6), but whether *BmABC009452 *and other silkworm ABCB genes are involved in xenobiotic resistances requires further investigation [10].

#### ABCC

The mammalian ABCC family members, including several multidrug resistance-associated proteins (MRP), are involved in ion transport, toxin secretion, signal transduction, and other physiological functions [5]. It has been suggested that the insect ABCC transporters might be important for xenobiotic resistance [10]. With 15 members in total, ABCC is the largest ABC subfamily in the silkworm. Moreover, there are 31 putative ABCC transporters in *T. castaneum*. Interestingly, 8 of the 15 silkworm ABCC genes are located on chromosome 15 and 4 on chromosome 12. Phylogenetic analysis of the ABCC subfamily within the 5 insect species reveals that many ABCC genes belonging to the same insect species cluster together, indicating gene duplication is an active mechanism that generates high diversity in the ABCC subfamily in insects (Additional File 7: Fig. S7). The silkworm ABCC subfamily consists of 5 full transporters, 7 half transporters, and 3 incomplete ABC proteins. All the 14 ABCC transporters in *D. melanogaster *are full transporters, whereas both full and half transporters exist in the ABCC subfamily of the four other insect species, supporting the evolutionary divergence of the ABCC subfamily in insects. The ABCC transporter encoded by *CG10505 *in *D. melanogaster *is regulated by heavy metals via the metal-responsive transcription factor 1 and involved in biochemical detoxification of zinc and copper [22]. Although several silkworm ABCC transporters have sequence similarity to the protein encoded by *CG10505 *in *D. melanogaster *(Additional File 7: Fig. S7), whether they might act as biochemical factors in the defense against toxins is unknown.

#### ABCD

The ABCD transporters are located to the peroxisome at the subcellular level and involved in the import of fatty acids and/or fatty acyl-CoAs into this organelle [23]. Each of the 5 insect species consists of 2 half transporters in the ABCD subfamily. The insect ABCD transporters are separated into two distinct groups by phylogenetic analysis (Additional File 8: Fig. S8), indicating that they are evolutionarily conserved in insects.

#### ABCE and ABCF

The ABCE and ABCF proteins lack TMD and each member contains a pair of linked NBDs [24]. Most eukaryotes have only one ABCE gene that is highly conserved during evolution (Additional File 9: Fig. S9). Different from most ABC transporters, their functions in cell biological processes are not related to transport. In human, ABCE1 was identified as an inhibitor of RNase L [25], whereas in yeast, ABCE proteins play key roles in translation initiation [26]. The silkworm ABCE gene, *BmABC010129 *which shows the highest expression level based on the EST information, is preferentially expressed in testis, ovary and fat body. However, RNA interference (RNAi)-mediated knockdown of *BmABC010129 *has no obvious effects on *RNase L *expression [27].

ABCF proteins function in ribosome biogenesis, translational control, and mRNA export, and are not involved in molecule transport [28]. The best-characterized ABCF protein is the yeast GCN20 protein, which is involved in the initiation and control of translation. In addition, mutations in *GCN20 *gene reduce eIF2α phosphorylation and thus translation in the ribosome [29]. Later studies revealed that ABCF1 physically interacts with eIF2 and associates with the ribosome in an ATP-dependent manner [28]. Until now, the ABCF subfamily has not been studied in insects. The same as human and most of other organisms, each of the 5 insect species consists of 3 ABCF proteins, which are separated into 3 distinct groups by phylogenetic analysis and genes in each group have high similarity (Additional File 9: Fig. S9). These data suggest that ABCE and ABCF subfamilies are highly conserved during evolution in all living organisms.

#### ABCG

The reported ABCG transporters are half transporters. Importantly, each ABCG transporter has a TMD at the C-terminal region of NBD, showing a distinct structure [1]. In human, members of the ABCG subfamily play key roles in lipid transport across membranes. For example, ABCG2 is an essential MDR and its activity is associated with decreased efficacy of anticancer agents in several carcinomas. Apart from its role in cancer, ABCG2 has a broad substrate specificity and its ability to transport numerous diverse pharmaceuticals has implications for the absorption, distribution, metabolism, excretion, and toxicity profile of these compounds. In addition, ABCG2 plays a role in the normal physiological transport of urate and haem [30]. The silkworm ABCG subfamily has 13 members, and 11 of them encode half transporters. Gene duplication is evident in this subfamily by the localization of gene paralogues or orthologs on chromosomes 5, 10, 12 and 22, which is further supported by phylogenetic analysis (Additional File 10: Fig. S10). The best-studied ABCG genes are those encoding eye pigment precursor transporters, *white*, *scarlet*, and *brown*, in *D. melanogaster *[11]. *BmABC002922 *(*Bmwh3*) encodes an ortholog of *D. melanogaster white*. Polyadenylated *Bmwh3 *transcript of about 2.7 kb length is detected in eggs, Malpighian tubule and pupal heads, but not in testes, posterior silk glands or fat body cells [31]. *Bmwh3 *is responsible for the transportation of ommochrome precursors and uric acid into pigment granules and urate granules, respectively, and *Bmwh3 *mutations cause white eyes, white eggs, and translucent larval skin [32]. *BmABC002924 *encodes an ortholog of *D. melanogaster scarlet*. In the silkworm, Scarlet forms a heterodimer with White to transport ommochrome precursors [33]. *BmABC002581 *encodes an ortholog of *D. melanogaster brown*. In addition, phylogenetic analysis revealed that *BmABC010557 *is orthologous to *D. melanogaster E23 *(*CG3327*) (Additional File 10: Fig. S10), which is a 20E primary response gene that represses 20E-mediated gene activation [12]. The potential physiological function of the silkworm ABCG subfamily is of interest.

#### ABCH

The ABCH subfamily was first identified in *D. melanogaster *and exists solely in arthropods [2]. The structure organizations of the ABCH and ABCG transporters are similar. Each of the 5 insect genomes studied in this report has 3 ABCH genes. Phylogenetic analysis suggests that the ABCH genes are originated from a common ancestor (Additional File 11: Fig. S11), yet their physiological functions remain unknown.

### Tissue distribution analysis reveals some tissue-specific ABC genes

**Figure 3:**
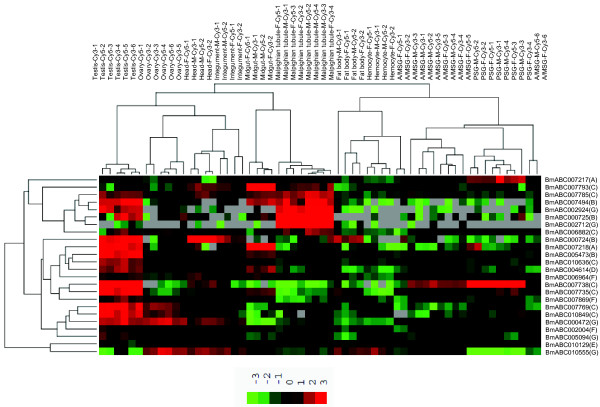
**Spatial expression patterns of the silkworm ABC genes in multiple larval tissues on day 3 of fifth instar**. Normalized microarray data (GSE17571) for gene expression in multiple tissues of silkworm larvae on day 3 of fifth instar was used to survey tissue-specific expression of ABC genes. ABC transporter gene was considered to be expressed if its normalized intensity value exceeded 0. The expression levels are illustrated by seven grade color scales representing relative expression levels of -3, -2, -1, 0, 1, 2 and 3. Red color represent positive; black color represent zero; green color represent negative; A/MSG anterior/middle silk gland, PSG posterior silk gland.

In order to understand the possible physiological function and hormonal regulation of the silkworm ABC genes in the future, we here analyzed the expression patterns of all the silkworm ABC genes by microarray data. With the exception of *BmABC010557*, all other ABC genes have corresponding probes in the oligonucleotide chip [34]. We first investigated the spatial expression patterns of the silkworm ABC genes on day 3 of fifth larval instar using the microarray data of tissue-specific expression [34]. The analysis revealed that the expression of 24 ABC genes can be detected at least in one of the selected tissues, including testis, ovary, head, integument, Malpighian tubule, fat body, haemocyte, anterior and middle silk gland, and posterior silk gland (Figure 3). Significantly, a large number of ABC genes were highly expressed in the testis compared to other tissues. It is known that more than one thousand genes are expressed specifically in the testis [34]. However, whether all these testis-specific ABC transporters are functionally important in this male organ is questionable and worthy of further investigation.

**Figure 4:**
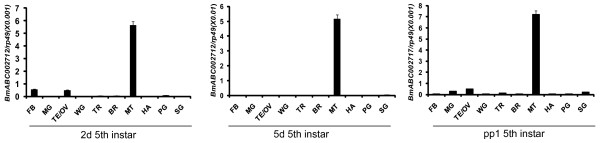
**Tissue distribution of *BmABC002712*, an ABCG gene, on day 2 and 5 of fifth larval instar and day 1 of prepupae**. FB (Fat body); MG (Midgut); TE/OV (Testes and ovaries); WG (Wing germ); TR (Trachea); BR (Brain); MT (Malpighian tubule); HA (Haemocytes); PG (Prothoracic gland); SG (Silk gland). *Rp49 *is used as an internal control. Relative mRNA level is indicated as the ratio of mRNA levels between the target gene and *Rp49*. Error bar represents SD of three independent replicates in this and all subsequent figures. In each replicate, ten animals were used and three times real time PCR analyses were conducted.

Some tissue-specific ABC genes were verified by qRT-PCR in nine tissues, including fat body, midgut, testis and ovary, wing disc, trachea, brain, Malpighian tubule, haemocyte, prothoracic gland, and silk gland, on day 2 and 5 of fifth larval instar and day 1 of prepupae. Besides the testis, the Malpighian tubule is the second tissue enriched with ABC gene expressions. The Malpighian tubule serves as the excretory and osmoregulatory organs for insects, in which the urine is produced and transported to the hindgut for the selective absorption of water and ions. Thus, the Malpighian tubule plays an important role in excretion and it is also involved in xenobiotic detoxification [35]. *BmABC002712*, an ABCG gene, was exclusively and abundantly expressed in the Malpighian tubule, detected by both microarray (Figure 3) and qRT-PCR (Figure 4), suggesting that *BmABC002712 *might play an important role in the Malpighian tubule. *BmABC002712 *could serve as a Malpighian tubule-specific molecule marker for future studies of gene function and regulation.

### Developmental expression analysis reveals several midgut-specific ABCG genes

**Figure 5:**
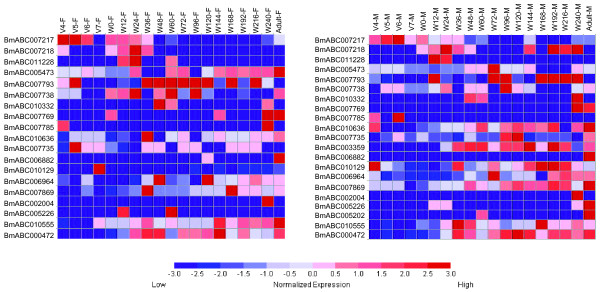
**Temporal expression patterns of the silkworm ABC genes during the larval-pupal-adult metamorphosis**. Female and male individuals at 19 time points during silkworm metamorphosis were selected for expression profiling using microarray analysis. The sequential time points include V4 (day 4 of the fifth larval instar), V5, V6, V7, W0 (0 h after wandering, just before spinning), W12, W24, W36, W48 (completion of spinning), W60 (immediately after pupation), W72, W96, W120, W144, W168, W192, W216, W240, and adult. V, fifth larval instar; W, wandering.

**Figure 6:**
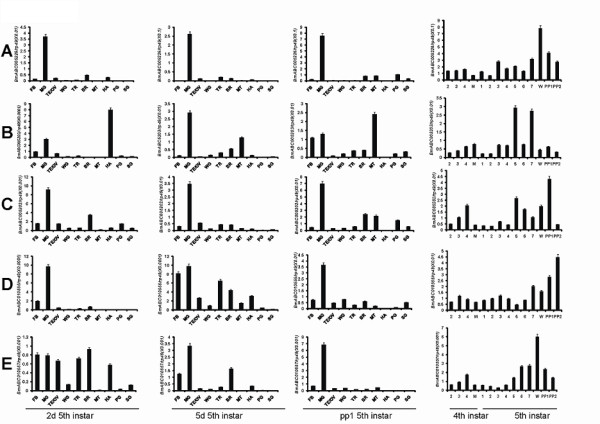
**Tissue distribution and developmental expression profiles of the 5 silkworm ABCG genes, including *BmABC005226, BmABC005203*, *BmABC005202, BmABC010555 *and *BmABC010557*, in the midgut from day 2 of 4^th ^instar to day 2 of prepupae**. M, molting; PP1, day 1 of prepupae; PP2, day 2 of prepupae.

In a microarray study published previously [13,14], we detected multiple silkworm ABC genes that are highly expressed during molting and pupation, exhibiting possible regulation by 20E. We then analyzed the temporal expression patterns of the silkworm ABC genes from day 4 of fifth instar to the adult stage using the microarray data of developmental changes of mRNA [34]. The analysis revealed that the expression of 19 and 21 ABC genes can be detected in females and males, respectively (Figure 5). We found that *BmABC005226*, an ABCG gene, was highly expressed during pupation (12 hours after the wandering stage) and the early pupal stage (60 hours after the wandering stage), when the 20E level is high. As determined by qRT-PCR, *BmABC005226 *is exclusively expressed in the midgut and highly expressed during the wandering and prepupal stages, when the 20E level is high (Figure 6A). The microarray and qRT-PCR data suggest that the midgut-specific ABCG gene, *BmABC005226*, is regulated by 20E at the transcriptional level.

We are very interested in the ABCG subfamily, which is particular in both structure and function [30]. In an unpublished survey in *D. melanogaster*, we have found that several ABCG transporters are exclusively expressed in two important endocrine organs, the corpus allatum which produces juvenile hormone and the prothoracic gland which produces ecdysone (the 20E immediate precursor). To this end, we investigated the spatial expression patterns of all the 13 silkworm ABCG genes using qRT-PCR. Besides *BmABC005226*, four other genes, including *BmABC005203*, *BmABC005202*, *BmABC010555*, and *BmABC010557*, are also preferentially expressed in the midgut. Moreover, we investigated their temporal expression patterns in the midgut, which revealed that the 4 ABCG genes are highly expressed during the wandering and prepupal stages as well. The qRT-PCR data (Figure 6B-E) roughly matches the microarray data (Figure 5), implying that they are also 20E responsive.

In addition, *BmABC002581 *(*brown*) and *BmABC012035 *were highly expressed in brain, and the mRNA level of *brown *peaked during the feeding stage of the fifth instar (Additional File 12: Fig. S12). Unfortunately, we did not find any prothoracic gland-specific ABCG genes in the silkworm.

### The midgut-specific ABCG genes were regulated by 20E via its receptor EcR-USP

**Figure 7:**
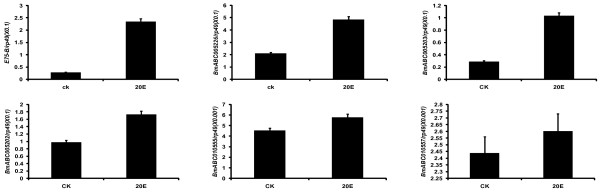
**Up-regulation of the 5 silkworm ABCG genes, including *BmABC005226, BmABC005203*, *BmABC005202, BmABC010555 *and *BmABC010557*, in the midgut by 20E treatment**. *E75 *was used as a positive control. Six hours after 20E treatment, larvae were sacrificed to dissect tissues for qRT-PCR analysis. Ten animals were used for each group and 3 biological replicates were conducted.

**Figure 8:**
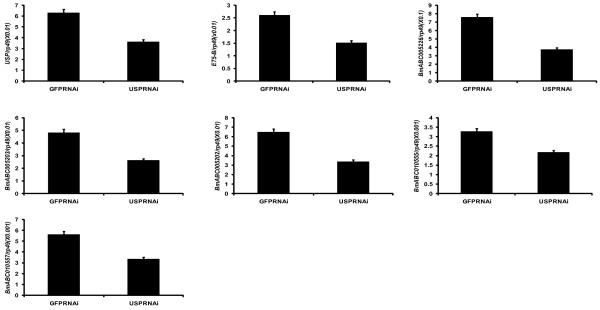
**Down-regulation of the 5 silkworm ABCG genes, including *BmABC005226, BmABC005203*, *BmABC005202, BmABC010555 *and *BmABC010557*, in the midgut by *USP *RNAi**. *E75 *was used as a positive control. Twenty four hours after RNAi treatment, the larvae were sacrificed for qRT-PCR analysis. Thirty animals were used for each group and 3 biological replicates were conducted.

The expression patterns in Figure 6 imply that the 5 midgut-specific ABCG genes, including *BmABC005226*, *BmABC005203*, *BmABC005202*, *BmABC010555*, and *BmABC010557*, might be up-regulated by 20E during molting and pupation. To test this hypothesis, we injected 20E into day 2 of fifth instar larvae and *USP *dsRNA into larvae at the initiation of the early wandering stage, followed by measurements of their mRNA levels using qRT-PCR [13,14]. As expected, the mRNA level of the 20E-primary response gene *E75 *was significantly up-regulated after 6 hours of 20E treatment and down-regulated after 24 hours of *USP *RNAi. Similarly, all the 6 ABCG genes were up-regulated by 20E treatment and down-regulated by *USP *RNAi (Figure 7 and 8), demonstrating that the 5 midgut-specific ABCG genes are up-regulated by 20E during molting and pupation.

It has been reported that the TMD of ABCG2 binds steroids and the binding mediates modulation of ABCG2 activity [36]. However, little is known about whether and how ABCG genes are transcriptionally regulated by steroid hormones in mammals. Since gene duplication is very evident in the ABCG subfamily in insects but not in human, the similarity of ABCG transporters in both physiological function and hormonal regulation is of great interest. We suppose that transcriptional regulation of ABCG genes by the steroid hormone 20E in the silkworm holds promising values for further investigation.

## Conclusion

This study provides a genome-wide analysis of the ABC transporters from the silkworm and a comparative analysis of the ABC family from 5 insect species. A large number of silkworm ABC genes are highly expressed in the testis and the Malpighian tubule. Two ABCG genes, *BmABC002712 *and *BmABC005226*, are exclusively expressed in the Malpighian tubule and the midgut, respectively. Moreover, 5 ABCG genes are preferentially expressed in the midgut and their transcriptional levels are up-regulated by 20E during molting and pupation. The descriptive study of silkworm ABCG genes lays a foundation for future analysis of their physiological function and hormonal regulation, using both the RNAi approach [13,14] and the binary GAL4/UAS transgenic system [37-39].

## Methods

### Animals

*Bombyx *larvae were provided by the Sericultural Research Institute, Chinese Academy of Agricultural Sciences. They were reared with fresh mulberry leaves in the laboratory at 25°C under 14 hour light/10 hour dark cycles [13,14].

### Gene identification, sequence alignment, phylogenetic analysis, and genomic distribution

We mainly used SilkDB [40,41] to search for potential ABC genes. First, we used the highly conserved NBDs of all 56 *D. melanogaster *ABC genes as queries to search against the updated GLEAN gene collection to identify silkworm ABC genes by local BLASTP [42], with an E-value threshold of 10^-6^. In addition, KAIKObase was also used, particularly for obtaining the full-length cDNA sequences. The identified putative ABC transporter genes were validated by search of the protein database of the NCBI with the putative ABC gene sequences as queries. Each potential ABC transporter was further analyzed by the program Pfam to identify its NBD and TMD domains. Finally, the identified silkworm ABC genes were used as queries to search SilkDB and KAIKObase again in order to avoid missing genes. The same methods were used for identification of ABC transporters in *A. gambiae*, *A. mellifera *and *T. castaneum*.

Protein sequence alignments were performed using ClustalX [43]. NBDs were then subjected to a phylogenetic analysis, using neighbor joining and bootstrapping with 1000 replicates in the program package MEGA4.0 [44].

We used SilkMap tool in silkworm genome database to map the loci of each ABC genes on the 28 chromosomes. Genes in clusters, for example, *BmABC000724 *and *BmABC000725 *on Chromosome 1, are indicated by a vertical line (Additional File 4: Fig. S4).

### Microarray analysis

We used silkworm genome-wide microarray data to profile the expression patterns of ABC genes in multiple larval tissues and during the larval-pupal-adult metamorphosis. The expression patterns of ABC transporter genes were estimated from intensity values [33]. From SilkDB, we downloaded normalized microarray data (GSE17571) for genome-wide gene expression in the anterior/middle silk gland (A/MSG), posterior silk gland (PSG), testis, ovary, fat body, midgut, integument, hemocyte, Malpighian tubule, and head on day 3 of the fifth larval instar. Normalized microarray data for gene expression at 19 time points during silkworm metamorphosis was recently performed (unpublished data from Southwest University). The sequential time points include V4 (day 4 of the fifth larval instar), V5, V6, V7, W0 (0 h after wandering), W12, W24, W36, W48, W60 (just after pupation), W72, W96, W120, W144, W168, W192, W216, W240, and adult (moth). The expression pattern of the ABC genes was estimated from intensity values. An ABC gene was considered to be expressed if its normalized intensity value exceeded 0 [34].

### qRT-PCR

Total RNA was extracted from the whole body or selected tissues of different developmental stages and used for qRT-PCR analysis as previously described [13,14]. Primers used here and somewhere else in this paper are listed in Additional File 13, Table S1.

### Hormone treatments

As described previously [13,14], day 2 of the fifth instar larvae (48 hrs after the fourth molting) was chosen for 20E injection (Sigma Aldrich, USA) (5 μg/larva) and the controls were injected with the same volume of control solvent. Six hours after 20E treatment, larvae were sacrificed to dissect tissues for qRT-PCR analysis. Ten animals were used for each group and 3 biological replicates were conducted.

### RNAi

dsRNA was generated using the T7 RiboMAX™ Express RNAi system (Promega, USA). At the initiation of the early wandering stage, each individual larva was injected with *GFP *dsRNA (10 μg) or *USP *dsRNA (10 μg). Twenty four hours after RNAi treatment, the larvae were sacrificed for qRT-PCR analysis [13,14]. Thirty animals were used for each group and 3 biological replicates were conducted.

## Authors' contributions

SL performed most of the experiments and analyzed the data. SZ, LT, EG helped with qRT-PCR, hormone treatment and RNAi. YL and JZ helped writing the paper and constructing the phylogenetic trees. SL designed the experiments, analyzed the data, wrote the paper, and coordinated the whole study. All authors approved the final manuscript.

## Supplementary Material

Additional file 1 **Figure S1**. Phylogenetic tree of the ABC transporters from the honeybee, *Apis mellifera*. Click here for file

Additional file 2 **Figure S2**. Phylogenetic tree of the ABC transporters from the mosquito, *Anopheles gambiae*. Click here for file

Additional file 3 **Figure S3**. Phylogenetic tree of the ABC transporters from the flour beetle, *Tribolium castaneum*. Click here for file

Additional file 4 **Figure S4**. Locations of the silkworm ABC genes on chromosomes. Genes in clusters, for example, *BmABC000724 *and *BmABC000725 *on Chromosome 1, are indicated by a vertical line. Click here for file

Additional file 5 **Figure S5**. Phylogenetic tree of ABCA transporters from five insect spcies and the human. The phylogenetic tree was constructed using a neighbor-joining technique to analyze the amino acid sequences of the nucleotide binding domain (NBD). Analysis was performed with the program package MEGA4.0. The number at the branch point of the node represents the value resulting from 1000 replications and gaps were deleted with pairwise deletion method. Am, *Apis mellifera*; Ag, *Anopheles gambiae*; Bm, *Bombyx mori*; Dm, *Drosophila melanogaster*; Hs, *Homo sapiens*; Tc, *Tribolium castaneum*. Click here for file

Additional file 6 **Figure S6**. Phylogenetic tree of ABCB transporters from five insect species and the human. Click here for file

Additional file 7 **Figure S7**. Phylogenetic tree of ABCC transporters from five insect species and the human. Click here for file

Additional file 8 **Figure S8**. Phylogenetic tree of ABCD transporters from five insect species and the human. Click here for file

Additional file 9 **Figure S9**. Phylogenetic tree of ABCE and ABCF proteins from five insect species and the human. Click here for file

Additional file 10 **Figure S10**. Phylogenetic tree of ABCG transporters from five insect species and the human. Click here for file

Additional file 11 **Figure S11**. Phylogenetic tree of ABCH transporters from five insect species and the human. Click here for file

Additional file 12 **Figure S12**. Tissue distribution and developmental expression profiles of the 2 silkworm ABCG genes, including *BmABC002581*and *BmABC012035 *in the brain from day 2 of 4^th ^instar to day 2 of prepupae. M, molting; PP1, day 1 of prepupae; PP2, day 2 of prepupae. Click here for file

Additional file 13 **Table S1**. A list of quantitative real-time PCR primers. Click here for file
