# Supplementary figures and images for: Genome-wide identification and characterization of ATP-binding cassette transporters in the silkworm, Bombyx mori
Source: BMC Genomics. 2011 Oct 7;12:491. doi: 10.1186/1471-2164-12-491 (PMC3224256; doi:10.1186/1471-2164-12-491)

## Figure S1

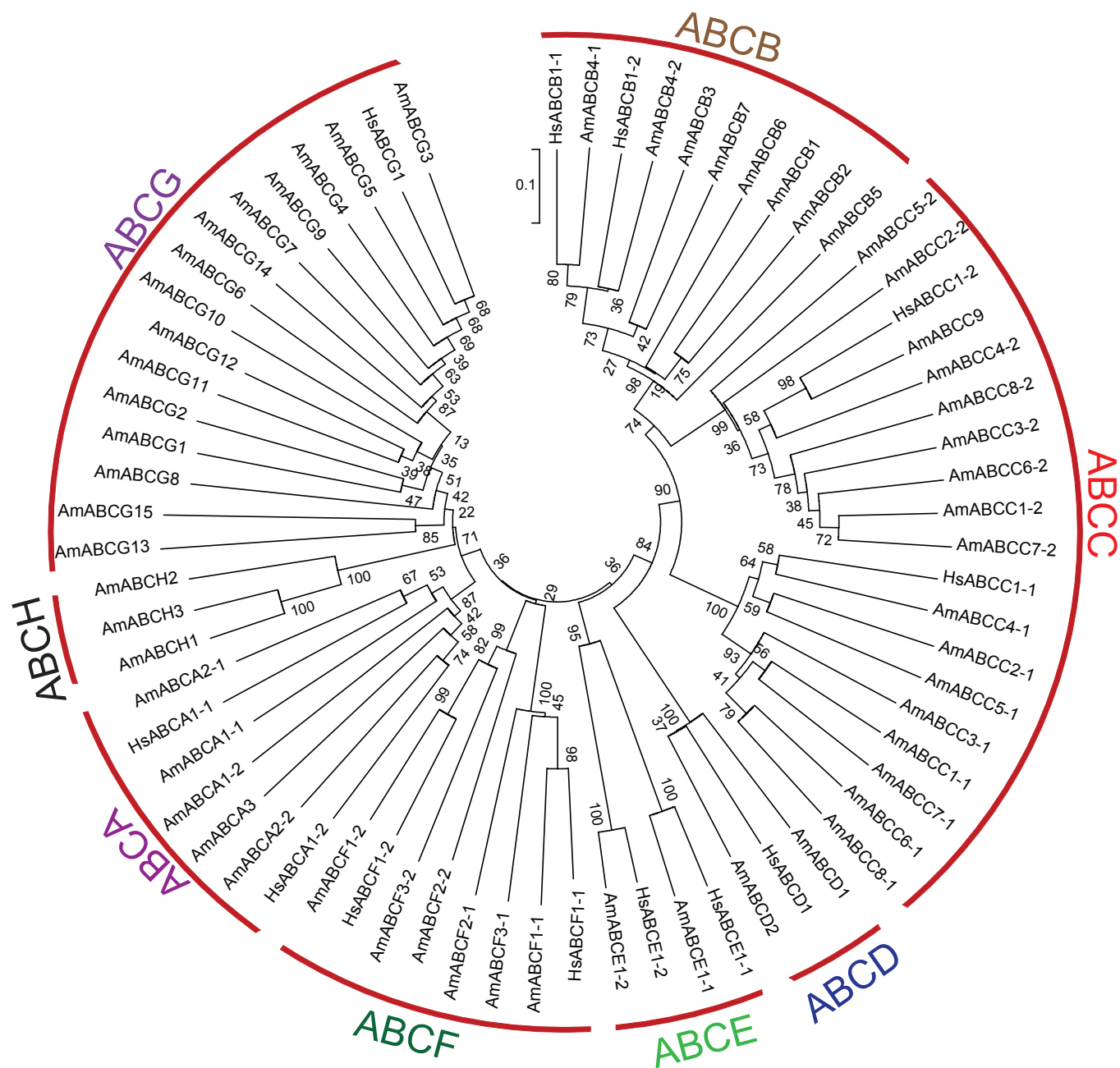

Supplement: Additional file 1 — Figure S1. Phylogenetic tree of the ABC transporters from the honeybee, Apis mellifera. [file 1471-2164-12-491-S1.PDF]

Figure S2

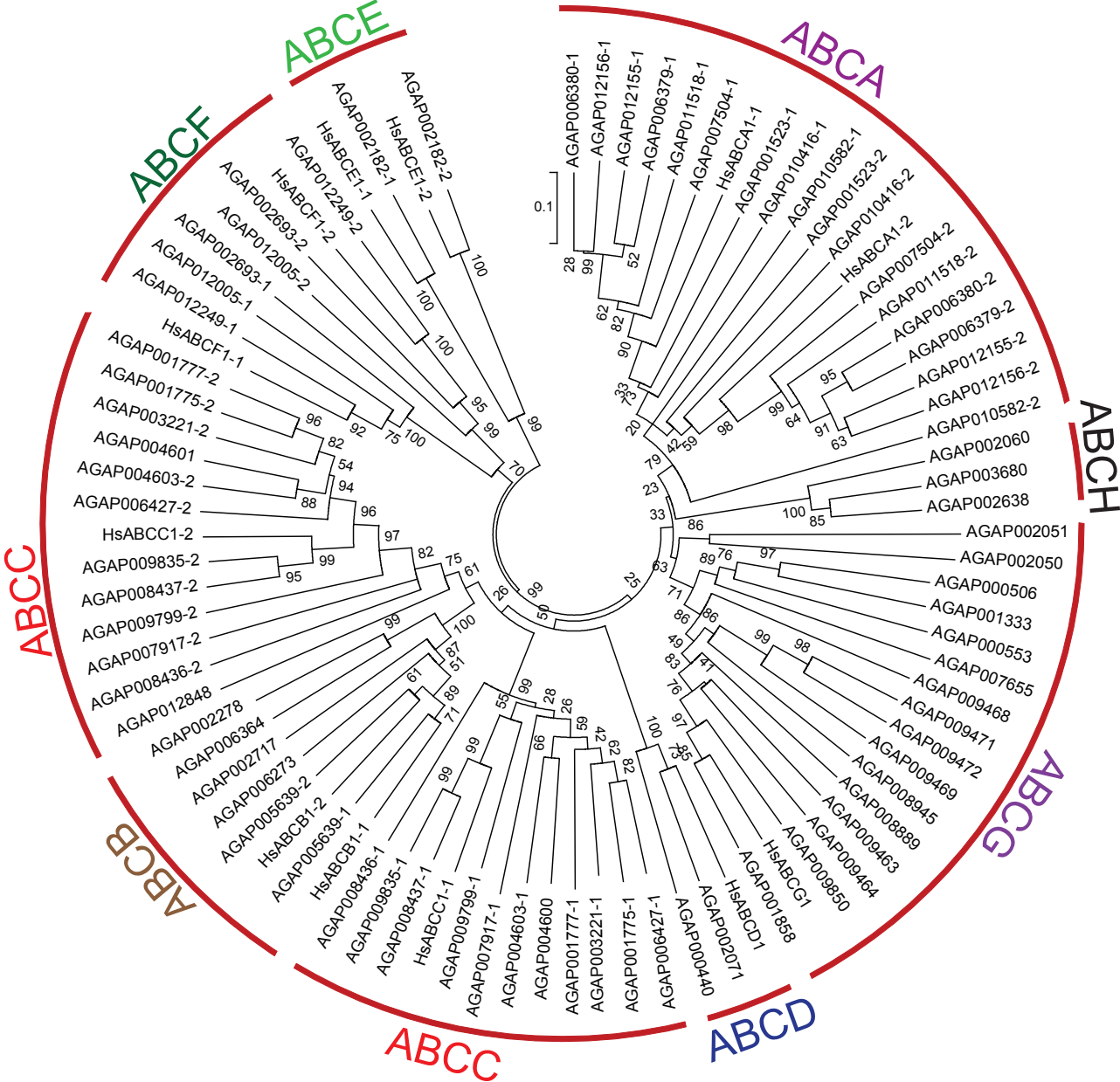

Supplement: Additional file 2 — Figure S2. Phylogenetic tree of the ABC transporters from the mosquito, Anopheles gambiae. [file 1471-2164-12-491-S2.PDF]

Figure S3

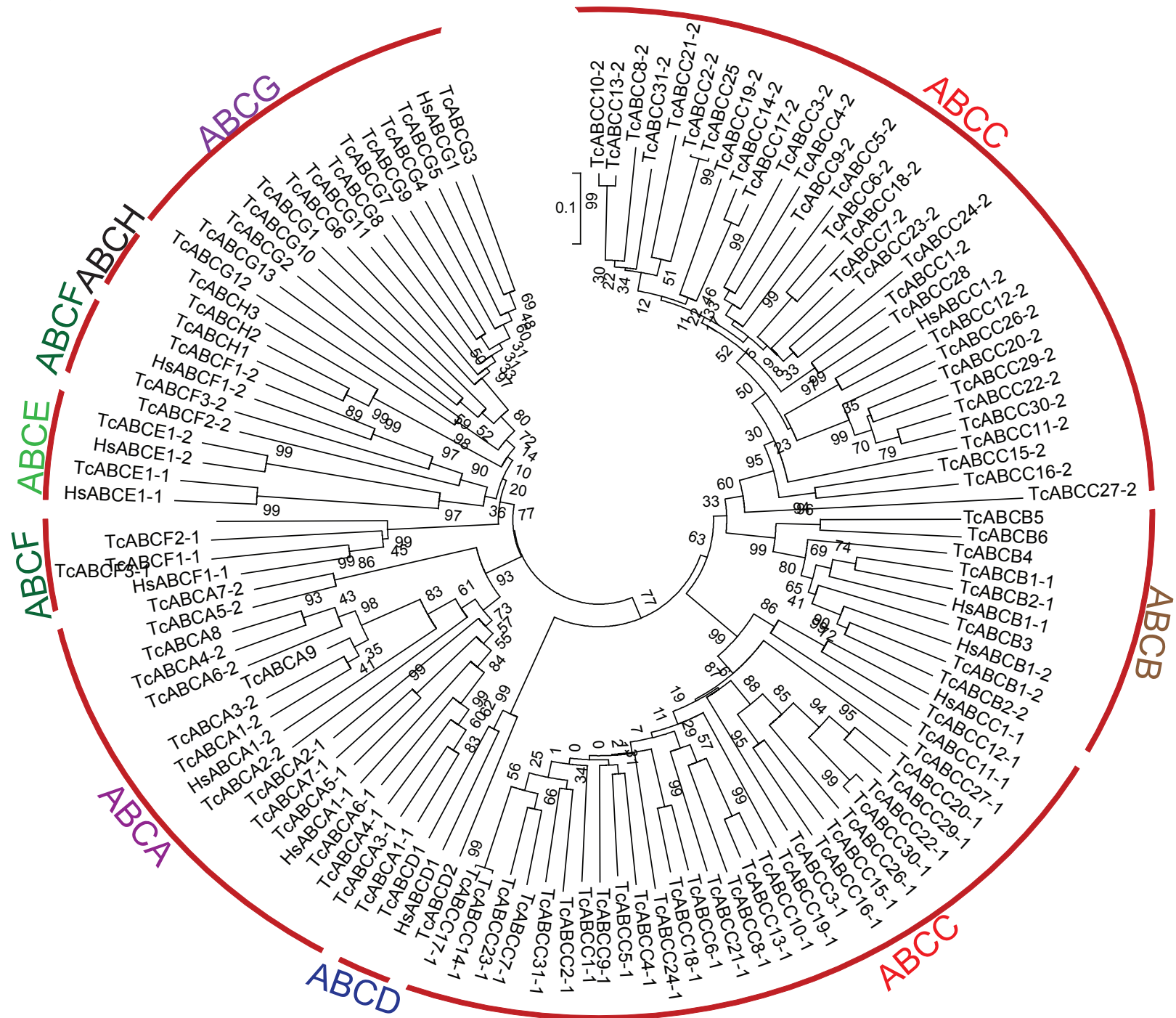

Supplement: Additional file 3 — Figure S3. Phylogenetic tree of the ABC transporters from the flour beetle, Tribolium castaneum. [file 1471-2164-12-491-S3.PDF]

Figure S4

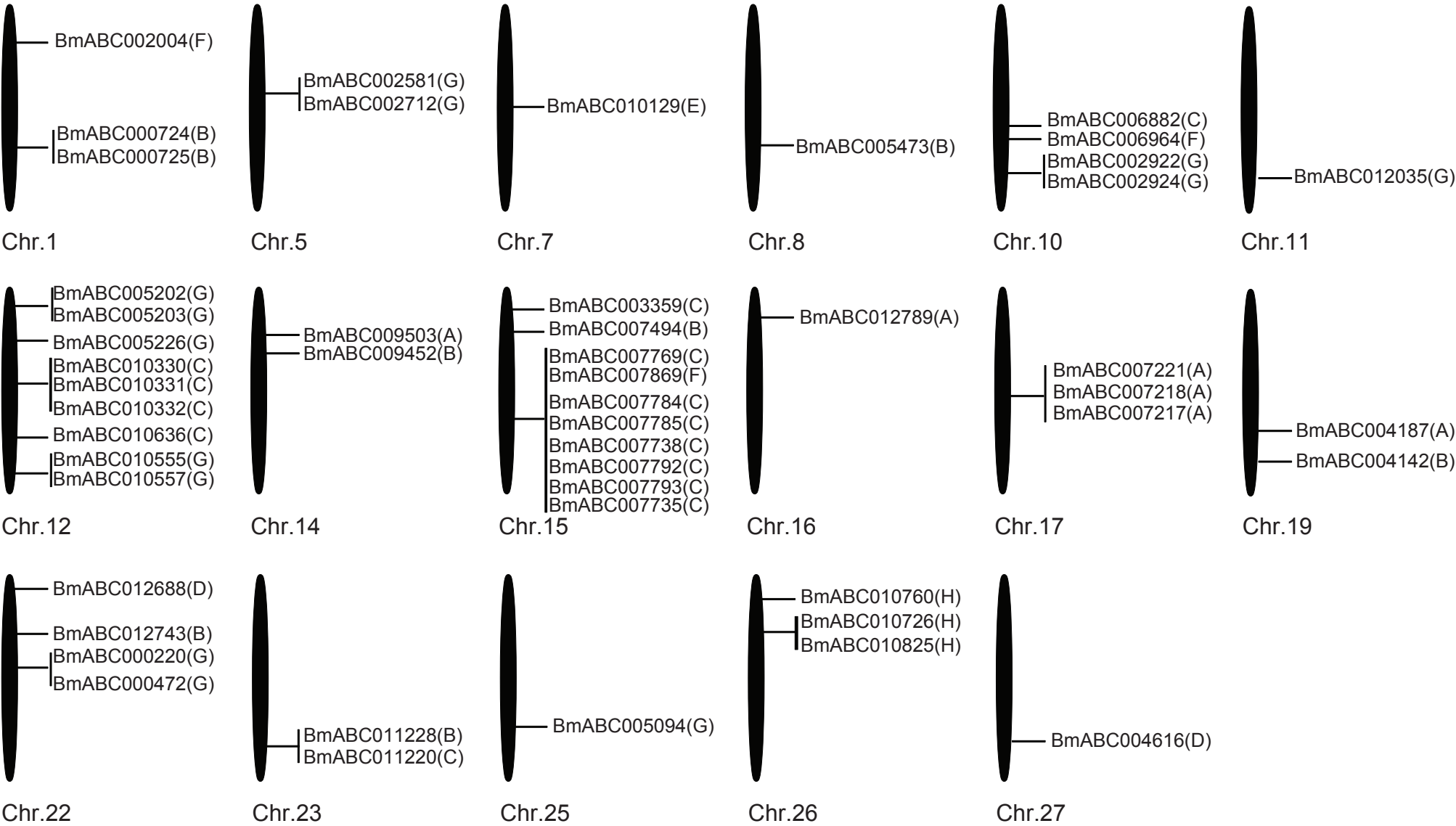

Supplement: Additional file 4 — Figure S4. Locations of the silkworm ABC genes on chromosomes. Genes in clusters, for example, BmABC000724 and BmABC000725 on Chromosome 1, are indicated by a vertical line. [file 1471-2164-12-491-S4.PDF]

## Figure S5

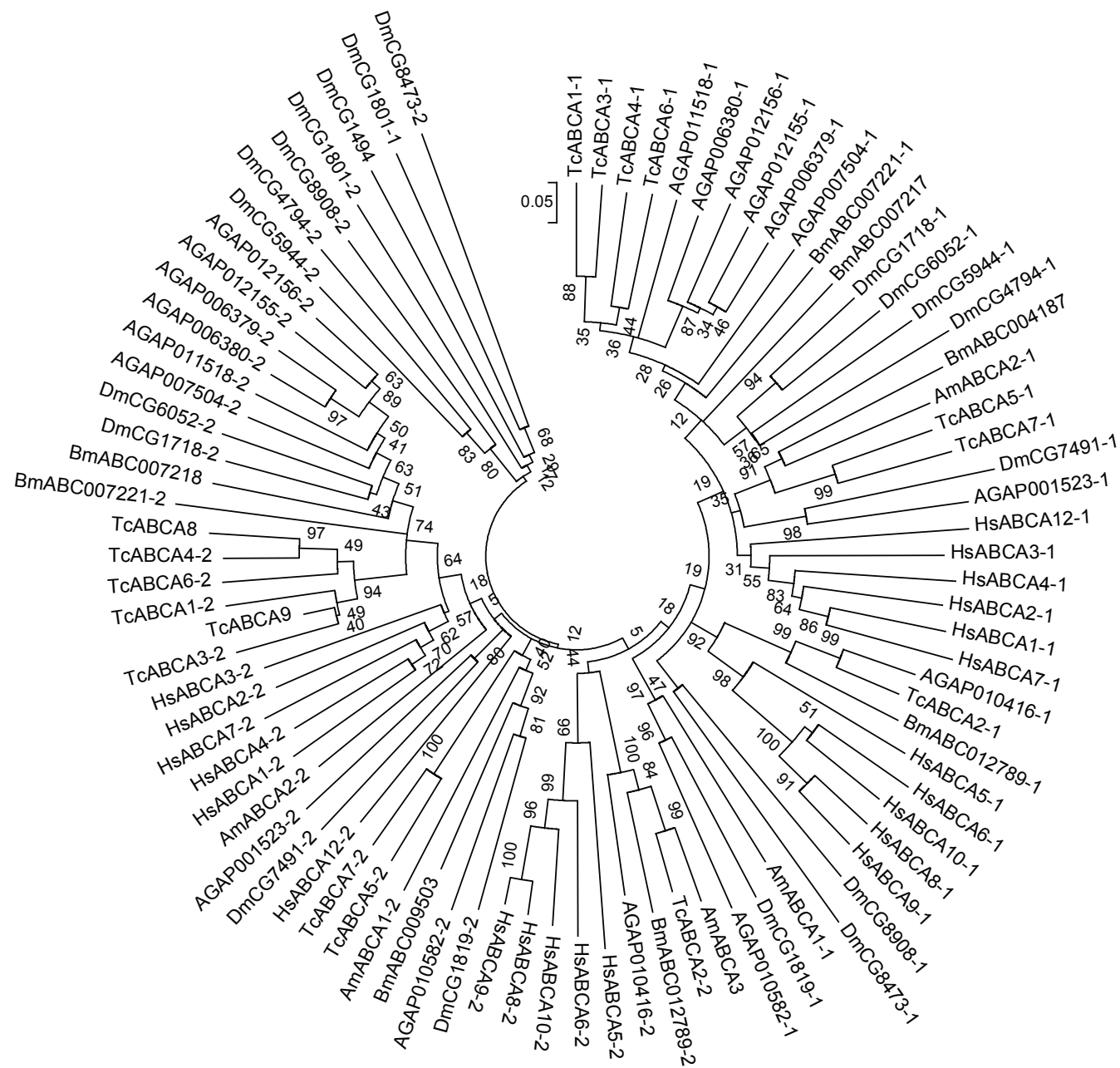

Supplement: Additional file 5 — Figure S5. Phylogenetic tree of ABCA transporters from five insect spcies and the human. The phylogenetic tree was constructed using a neighbor-joining technique to analyze the amino acid sequences of the nucleotide binding domain (NBD). Analysis was performed with the program package MEGA4.0. The number at the branch point of the node represents the value resulting from 1000 replications and gaps were deleted with pairwise deletion method. Am, Apis mellifera; Ag, Anopheles gambiae; Bm, Bombyx mori; Dm, Drosophila melanogaster; Hs, Homo sapiens; Tc, Tribolium castaneum. [file 1471-2164-12-491-S5.PDF]

Figure S6

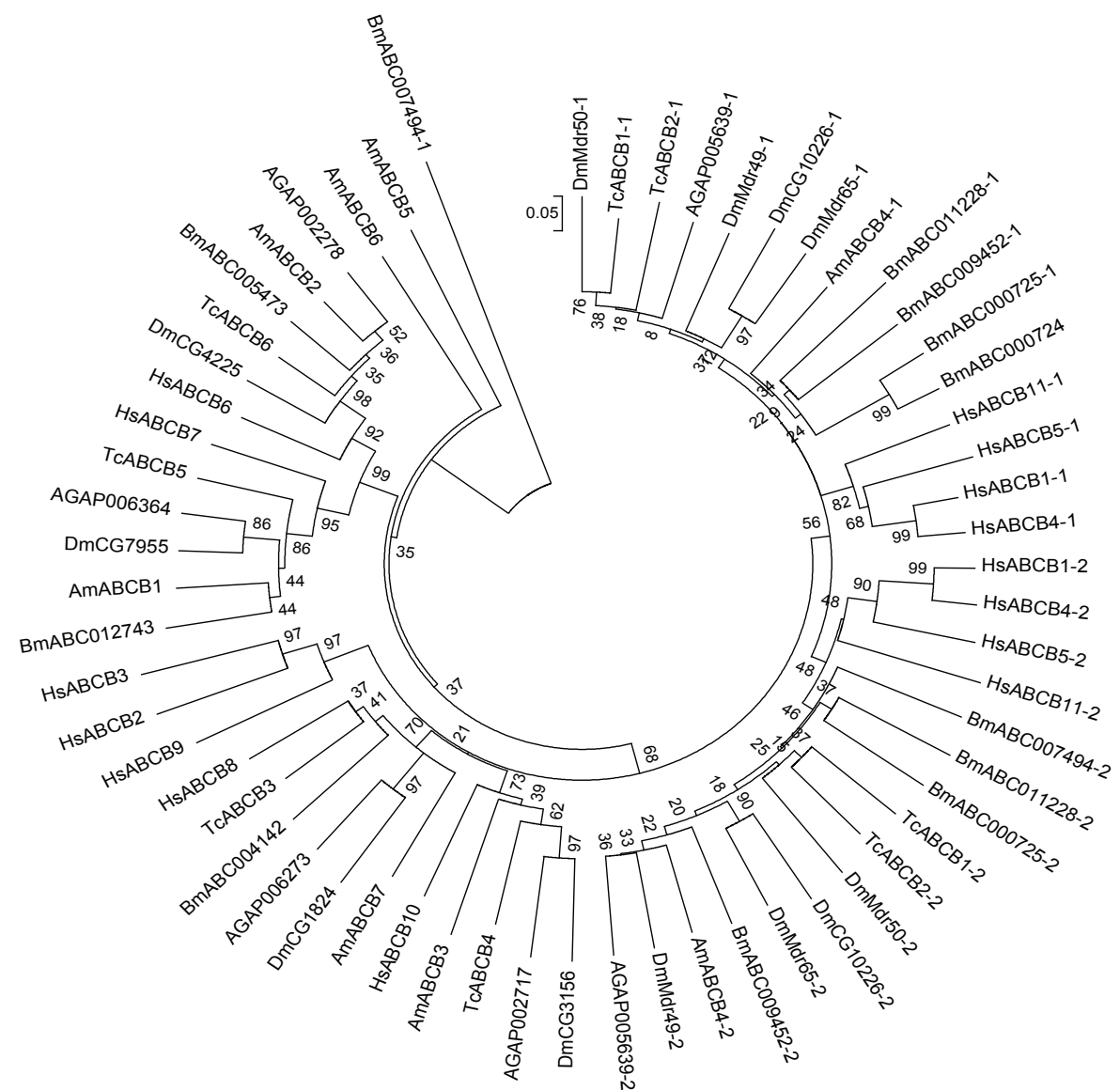

Supplement: Additional file 6 — Figure S6. Phylogenetic tree of ABCB transporters from five insect species and the human. [file 1471-2164-12-491-S6.PDF]

## Figure S7

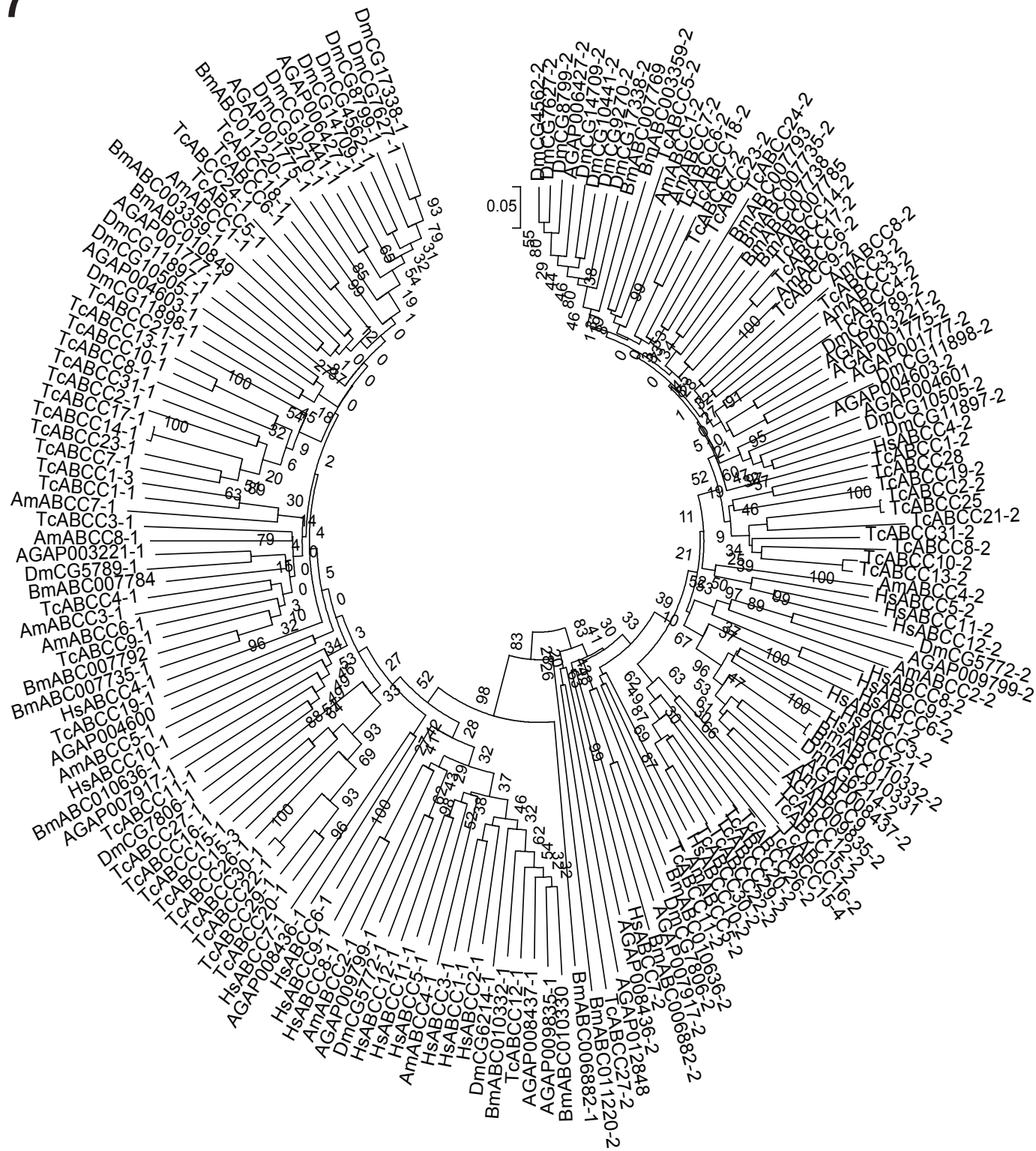

Supplement: Additional file 7 — Figure S7. Phylogenetic tree of ABCC transporters from five insect species and the human. [file 1471-2164-12-491-S7.PDF]

Figure S8

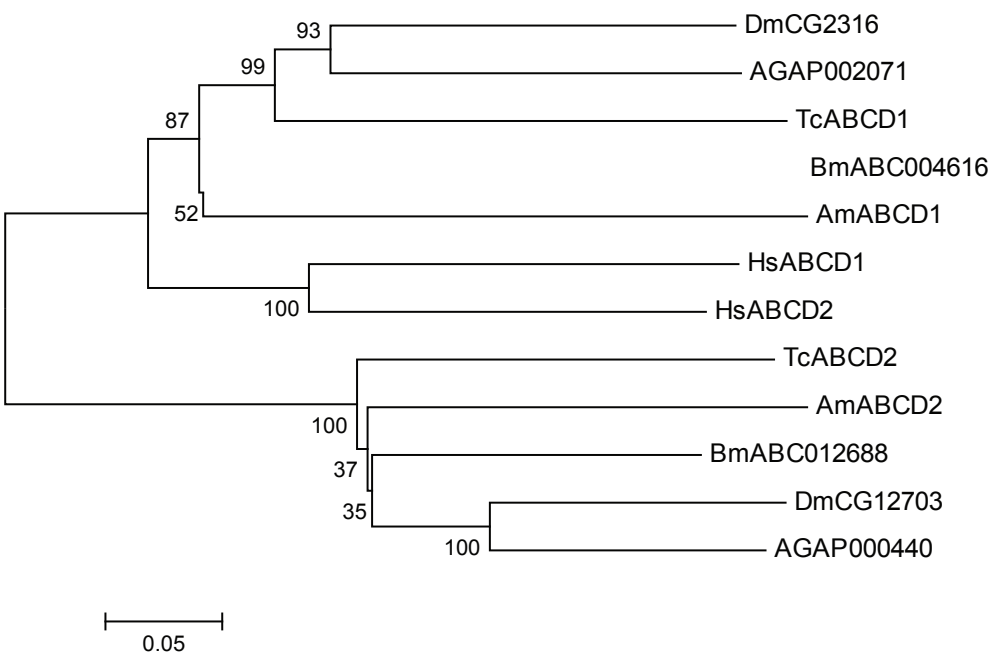

Supplement: Additional file 8 — Figure S8. Phylogenetic tree of ABCD transporters from five insect species and the human. [file 1471-2164-12-491-S8.PDF]

Figure S9

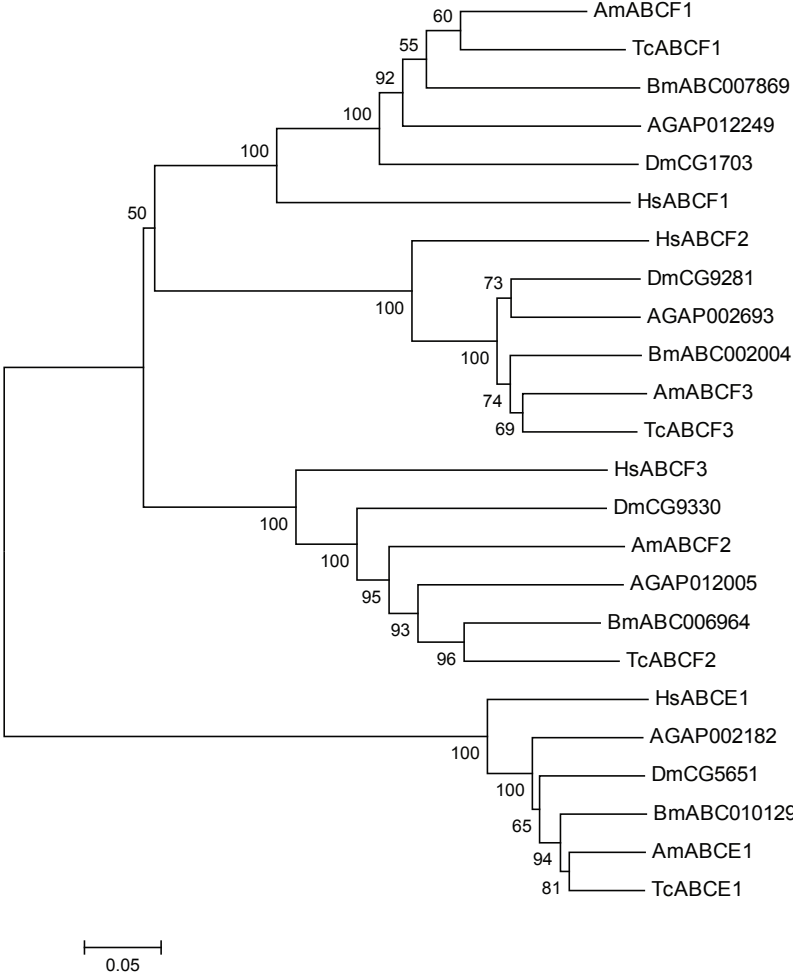

Supplement: Additional file 9 — Figure S9. Phylogenetic tree of ABCE and ABCF proteins from five insect species and the human. [file 1471-2164-12-491-S9.PDF]

Figure S10

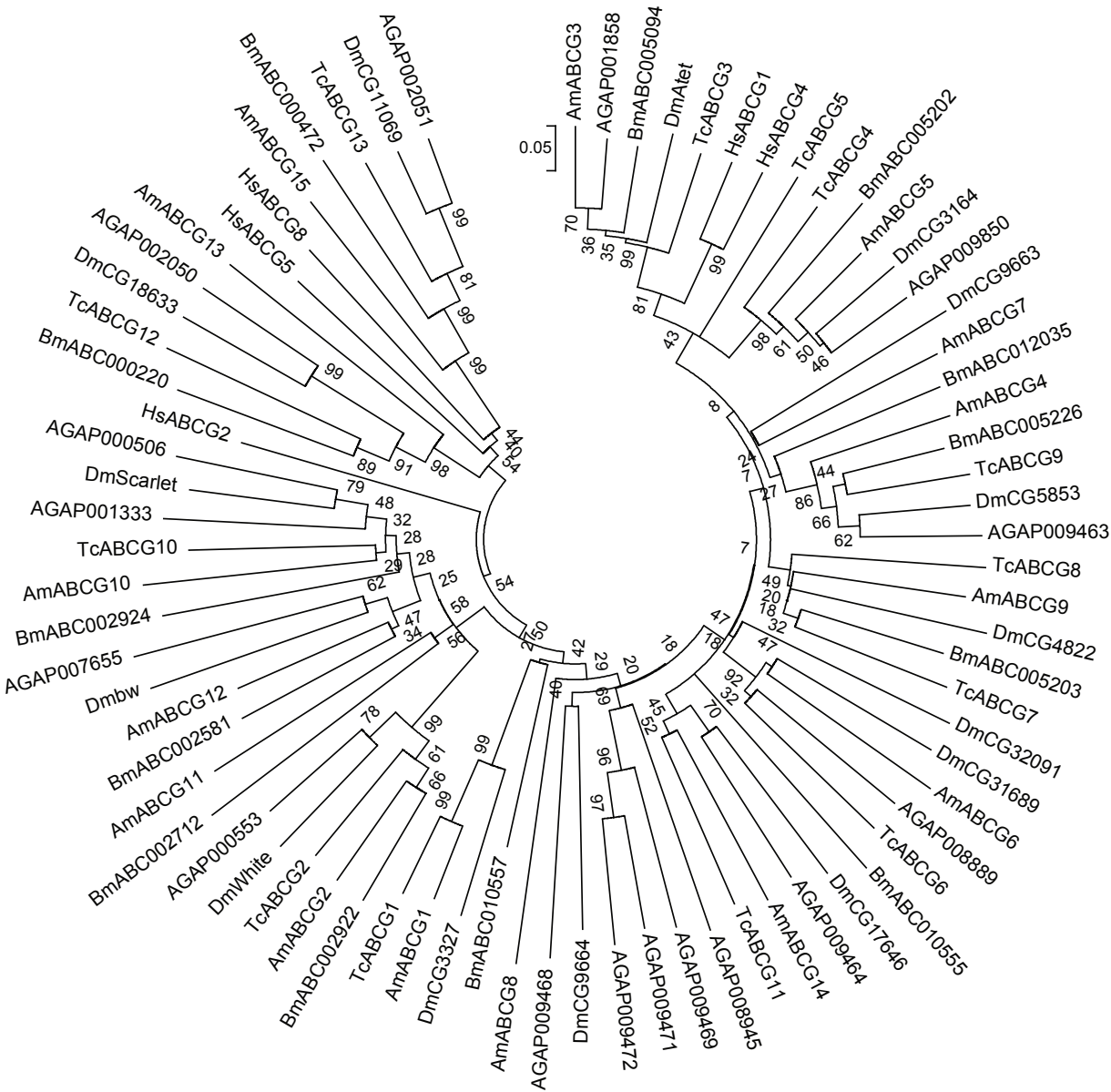

Supplement: Additional file 10 — Figure S10. Phylogenetic tree of ABCG transporters from five insect species and the human. [file 1471-2164-12-491-S10.PDF]

Figure S11

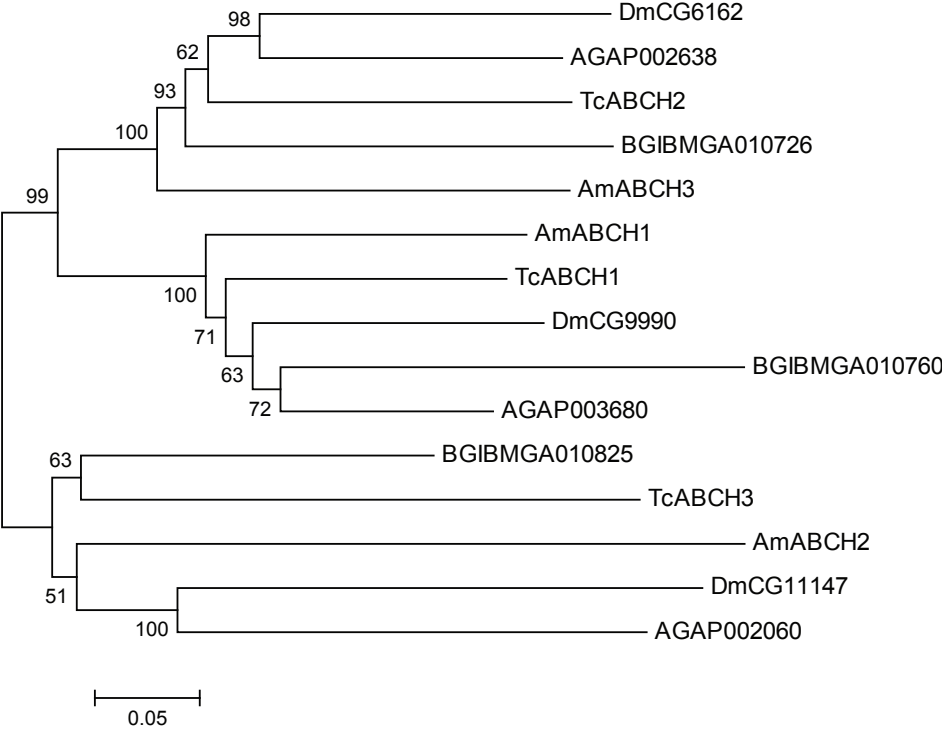

Supplement: Additional file 11 — Figure S11. Phylogenetic tree of ABCH transporters from five insect species and the human. [file 1471-2164-12-491-S11.PDF]

# Figure S12

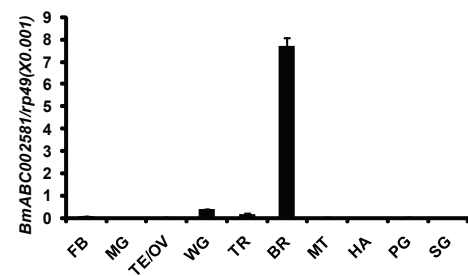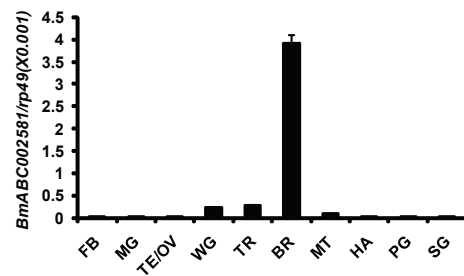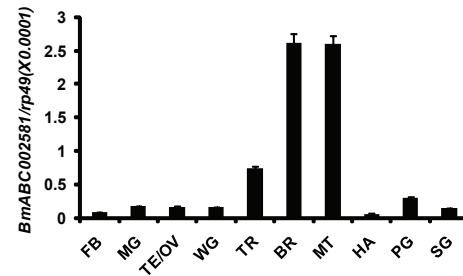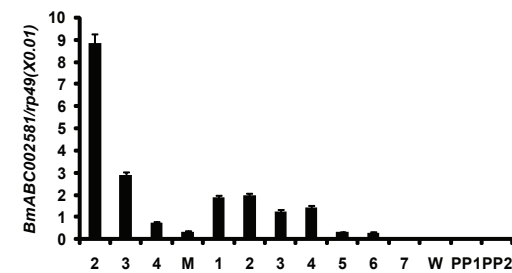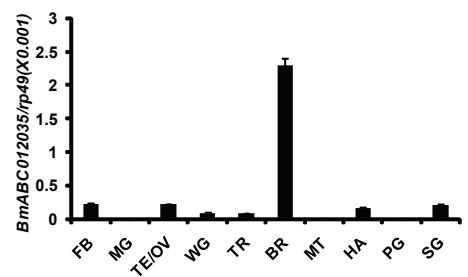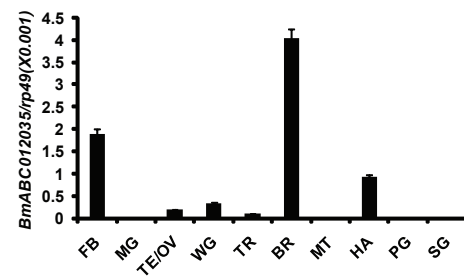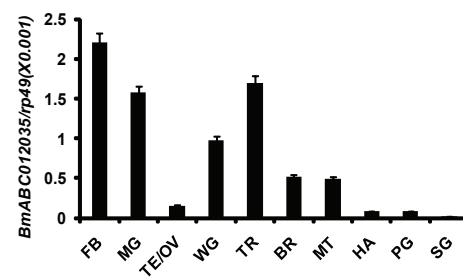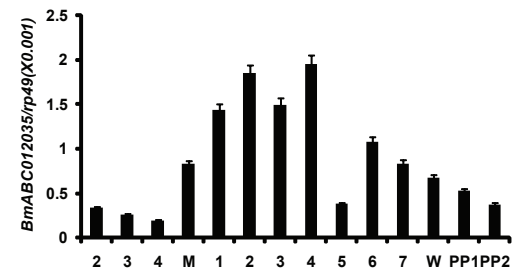

2d 5th instar

5d 5th instar

pp1 5th instar

4th instar

5th instar

Supplement: Additional file 12 — Figure S12. Tissue distribution and developmental expression profiles of the 2 silkworm ABCG genes, including BmABC002581and BmABC012035 in the brain from day 2 of 4th instar to day 2 of prepupae. M, molting; PP1, day 1 of prepupae; PP2, day 2 of prepupae. [file 1471-2164-12-491-S12.PDF]
