# Supplementary material for: Genome-wide identification and characterization of ATP-binding cassette transporters in the silkworm, Bombyx mori
Source: BMC Genomics. 2011 Oct 7;12:491. doi: 10.1186/1471-2164-12-491 (PMC3224256; doi:10.1186/1471-2164-12-491)
Supplement: Additional file 13 — Table S1. A list of quantitative real-time PCR primers. [file 1471-2164-12-491-S13.PDF]

Table S1

Gene Name	Forward Primer(5'-3')	Reverse Primer(5'-3')	Product Length
BmABC005226	ACTGGACTGCAATGAAGCAC	GCTGCAATCCTTAACACGAA	138
BmABC005203	GTTCCATTCCTCAACGACCT	CTCCATTTCTCGCTCTTTCC	146
BmABC005202	AAACGTCTCTCCATCGCTCT	ATGCATTGGAAACACGAAGA	101
BmABC010555	TCCGGTGAATACGACGTAGA	AAGAAGAAGGCAGTGCTGGT	121
BmABC010557	TAGGAATGGCGTGGACAATA	GATGAACAGTCCGAGCTTCA	101
BmABC011228	CTCGAAGGCTACGTGAACAA	TCCAAGACTGTCAGCTCCAC	134
BmABC010332	ATGGCTTGGTTTCCGATTAG	GGTTAAGGAAAGTCCCACGA	115
BmABC002581	CCCAGTGAGGTTCCCATACT	ATGAGGGAACCGAACAGAAC	131
BmABC012035	TATCCGGTTATCGTTCAGCA	TCTCGGACAGTGAGAAGTGG	136
BmABC010726	GAAGGAGCCACGATATCCAT	GCTGCCTCCTAGAAGCAACT	139
